# Towards New Horizons: Climate Trends in Europe Increase the Environmental Suitability for Permanent Populations of *Hyalomma marginatum* (Ixodidae)

**DOI:** 10.3390/pathogens10020095

**Published:** 2021-01-21

**Authors:** Natalia Fernández-Ruiz, Agustín Estrada-Peña

**Affiliations:** 1 Faculty of Veterinary Medicine, University of Zaragoza, 50013 Zaragoza, Spain; antricola@me.com; 2 Group of Research on Emerging Zoonoses, Instituto Agroalimentario de Aragón (IA2), 50013 Zaragoza, Spain

**Keywords:** *Hyalomma marginatum*, Europe, climate trends, environmental suitability

## Abstract

Ticks and tick-borne pathogens are changing their current distribution, presumably due to the impact of the climate trends. On a large scale, these trends are changing the environmental suitability of *Hyalomma marginatum,* the main vector of several pathogens affecting human health. We generated annual models of environmental suitability for the tick in the period 1970–2018, using harmonic regression-derived data of the daily maximum and minimum temperature, soil moisture and water vapor deficit. The results demonstrate an expansion of the suitable area in Mediterranean countries, southeast central Europe and south of the Balkans. Also, the models allowed us to interpret the impact of the ecological variables on these changes. We deduced that (i) maximum temperature was significant for all of the biogeographical categories, (ii) soil humidity has an influence in the Mediterranean climate areas, and (iii) the minimum temperature and deficit water vapor did not influence the environmental suitability of the species. The conclusions clearly show that climate change could create new areas in Europe with suitable climates for *H. marginatum*, while keeping its “historical” distribution in the Mediterranean. Therefore, it is necessary to further explore possible risk areas for *H. marginatum* and its associated pathogens.

## 1. Introduction

Major changes in ecosystems are being driven by the fast anthropogenic climate change observed in the early 21st Century [1]. Climate change is defined as “a statistical phenomenon that describes the average climatic conditions for a region, referred to systematic and generally gradual changes in the trend, which is integrated into the random fluctuations of the climate” [2]. Since empirical observations of weather are scattered over time, and memory of past events may be incomplete, climate change is not captured through personal experience. Climate change affects the dynamics of many vector-borne pathogens [3], their reservoirs and human habits, to a yet unknown scale. This may result in the (re)emergence of vector-borne diseases. While studies are diverse, probably the most easily captured effect is the alteration of the known distribution of health threatening pathogens transmitted by arthropods [4].

Ticks transmit the largest number of zoonotic agents in the Northern Hemisphere [5], and as such, are often the focus of research regarding the impact of climate change on human health. Ticks are hematophagous parasites that depend on an environmental niche of temperature and humidity ranges for their population to perpetuate; the overlap of this niche with the one preferred by many vertebrate reservoirs results in areas in which pathogens can circulate. Changes of the geographical distribution of ticks have been well documented for several species in the Nearctic [6,7,8] and Palearctic regions [9,10]. In short, most studies have connected the spread of ticks with warmer temperatures [11]. Interestingly, no reports seem to exist about the “decolonization” of ticks from their southern habitat as a potential consequence of environmental conditions becoming too warm or dry. 

Other than continuous spread by adjacency of populations at the fringe of their environmental niches, ticks can also be introduced into a territory by the uncontrolled movements of livestock or the seasonal behavior of migratory birds. Since the pioneering work by Hoogstraal and his team [12], it has been known that the migratory routes between Africa and Europe serve as routes of dissemination of ticks belonging to the genus *Hyalomma*. Birds carry immatures of at least two species of the genus (*Hyalomma marginatum* and *Hyalomma rufipes*) that feed for several days on hosts, thus allowing transportation and further spread into northern territories. For permanent populations of *Hyalomma* to establish, it is believed that a critical threshold of temperature is necessary for tick development; this temperature provides an index of the chance of establishment. In the second decade of the 20th century, it was believed that this was a rare event, because the critical temperature threshold for molt was not reached out of the Mediterranean range of the species [13]. However, reports of adult *Hyalomma* ticks in Europe [14,15,16,17,18] are becoming increasingly common, suggesting that the accelerated warming affecting central Europe could impact the development of molting immature ticks, allowing adults to survive in sites where they were historically absent. A previous study based on a model of the physiological processes of the tick [19] demonstrated that the climate changes which occurred during 20th century promoted changes in the developmental rates of *H. marginatum*. This tick is considered a concern for human health, because *Hyalomma* ticks are known vectors of the Crimean–Congo hemorrhagic fever virus and *Rickettsia aeschlimannii.* The former is an arbovirus belonging to the genus *Orthonairovirus* (family Bunyaviridae) [20]. Infected patients develop a hemorrhagic fever that has a lethality ranging between 3%–50%, depending on the strain [21]. The viral agent can produce sporadic cases [22] or severe outbreaks in a large geographic area, from western China to the Middle East and in the most southeastern region of Europe [23] and most of Africa [24]. Additionally, *Rickettsia aeschlimannii* is an intracellular bacterium included in the spotted fever group [25] that is of emerging importance in regions of Europe [26]).

This study focuses on changes induced by the climate since 1970, on the expected environmental suitability (ES) for *H. marginatum* in Europe. The aim is to address the joint changes of several weather variables affecting the life cycle of the tick, their trends and their effects on the expected range of its adequate environment. We extracted the daily maximum and minimum temperature, soil surface and air water vapor deficit for each year in the target period using harmonic regressions of a new generation of re-assessed climate data. We used the largest existing dataset of records of *H. marginatum* to develop models addressing the trend of ES for the tick, and produced multiple regressions against environmental stressors to determine the variables driving the suitability for the tick according to standard bio-geographical regions of Europe.

## 2. Results

**Figure 1:**
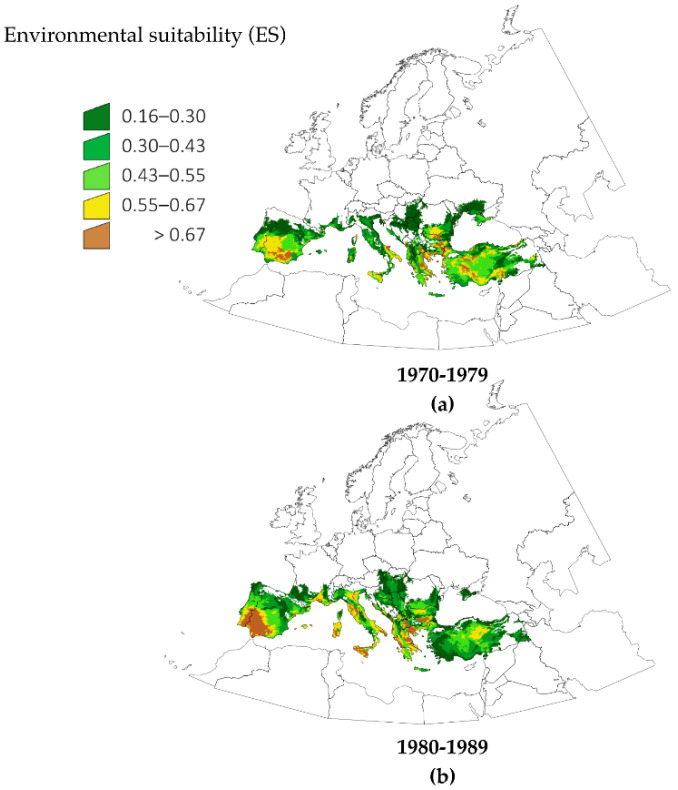

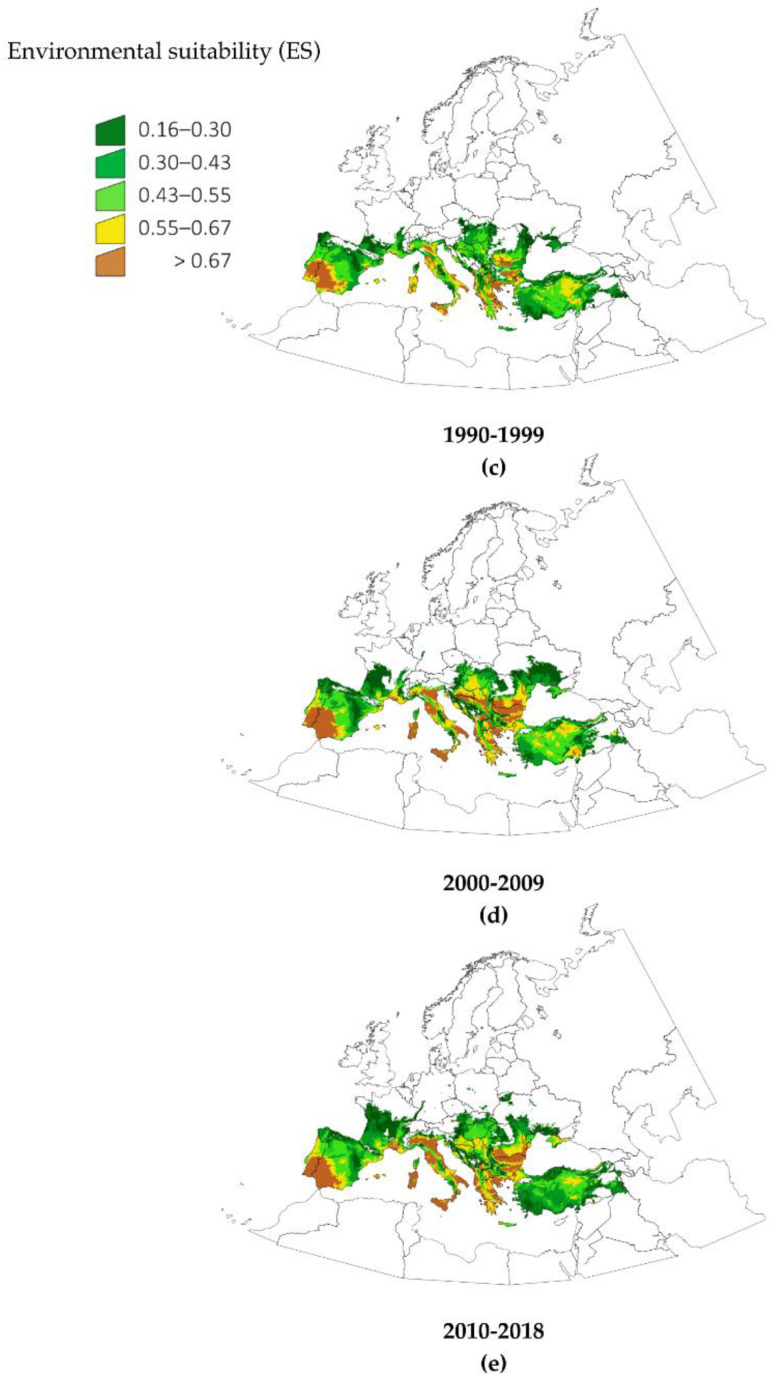
Spatial changes of environmental suitability for *Hyalomma marginatum* in five time slices covering periods of 10 consecutive years. (**a**) 1970–1979, (**b**) 1980–1989, (**c**) 1990–1999, (**d**) 2000–2009, (**e**) 2010–2018.

Figure 1 shows the calculated changes of ES for *H. marginatum* in five time slices of 10 years for the period 1970–2018. These predictive maps are built with a combination of climate variables and the actual records of the tick in the period 1990–2006 (see Methods for a complete description of the modeling process). The figure illustrates the changes of ES in time chunks that are adequate for illustrating the spatial patterns of variation. Notable changes occurred in south coastal and near inner parts of France, with an increase of suitable area in Italy, southeastern central Europe and southern Balkans. A detailed analysis of these data showed the unexpected result that changes had occurred not only in the fringe of the distribution of *H. marginatum*, but also in zones in the currently known distribution of the tick. The increase of ES was continuous throughout the time period for large regions in Spain, France, Italy and the Aegean coasts. The obvious increase of suitable conditions in the south-western Iberian Peninsula observed between 1970–1979 (Figure 1a) and 1980–1989 (Figure 1b) is particularly striking. The period 1990–1999 (Figure 1c) shows the increase of ES in parts of central Europe, Crimean Peninsula and northeast Turkey. The complete picture indicates that, for the period 2010–2018, the most drastic changes occurred in portions of the Mediterranean coast, some large areas around southeastern central Europe and the south Balkans. Results regarding central Europe and Balkans countries are of special interest in the context of the spread of *H. marginatum*.

**Figure 2:**
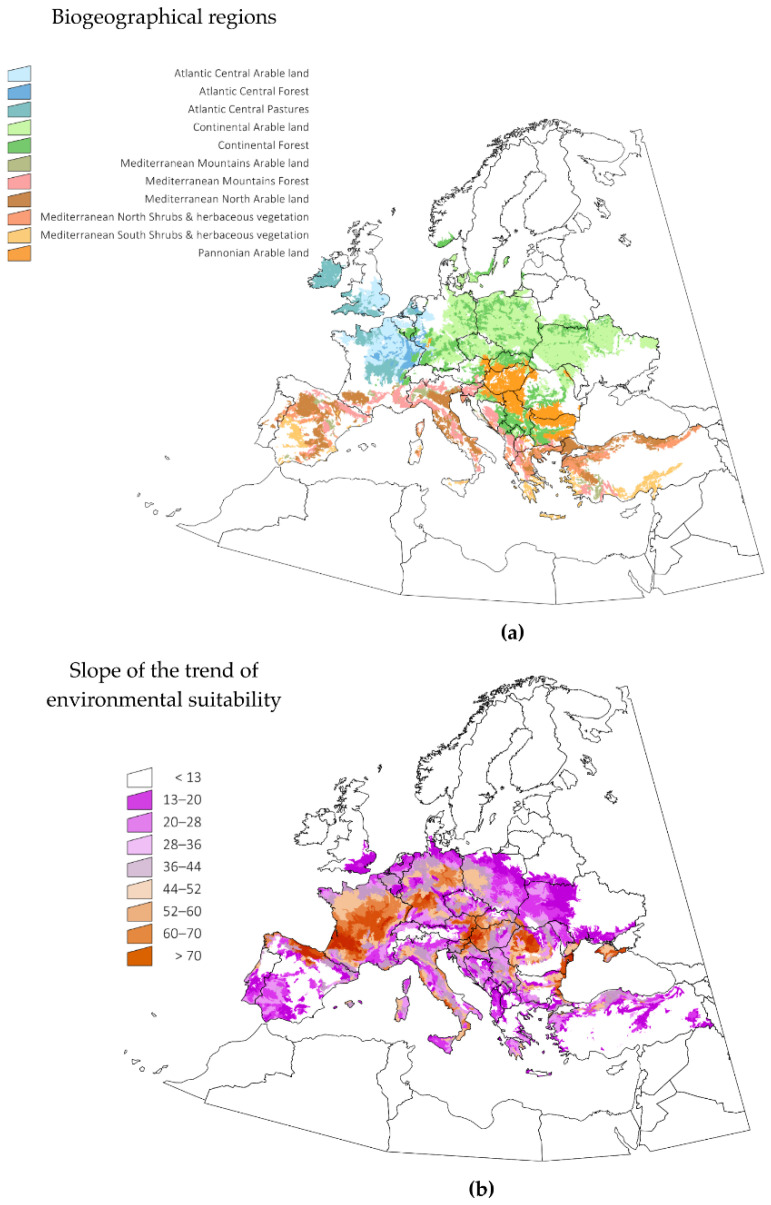
The slope of the changes of environmental suitability for *Hyalomma marginatum* in the target territory in the period 1970–2018. A positive slope is not correlated with a suitable environmental for the tick (see Figure 1 for this data), but rather, only the strength of changes of the environmental variables in the period of reference. These changes are shown in (**b**) with explicit reference to the biogeographic divisions in (**a**).

Figure 2 displays the changes in the trend of ES observed in selected LANMAP2 biogeographic regions of Europe, covering approximately the 80% of the territory (a selection of regions was made to improve the clarity of the presentation of this data). LANMAP2 is a general description of the biogeographic regions of Europe, and therefore, the reporting of the calculated changes according to landscape descriptions is of interest. The trend of ES was calculated by a lineal regression of its annual values in the period 1970–2018. The slope of the ES throughout this period was positive for every territory. It is noteworthy that the areas with the highest trend of ES were not those where *H. marginatum* was already established, but rather, those that are most likely to see future spread, because the environmental niche is quickly becoming suitable. Figure 2 displays the spatial trend of that slope. Major increases of ES for the tick resulted in areas of Lusitanian type climate, associated with landscape categories of pastures and permanent crops. Pannonian-, Atlantic central- and Continental-type climates experienced drastic increases in ES. A large increase in ES was predicted for Mediterranean mountains, suggesting a spread of the suitable environment of the tick into higher altitudes. Changes in climate variables are pushing *H. marginatum* to find adequate habitats further north and at higher altitudes. Interestingly, these changes are observed in different regions of Europe, meaning either an increase of ES in areas where the tick is already present or a trend toward suitability in sites where the ticks does not yet present permanent populations. It should be stressed that the presented ES trend is not an evaluation of the places that could be colonized by *H. marginatum*, but only a representation of the speed of change of climate traits relative to its environmental suitability.

**Figure 3:**
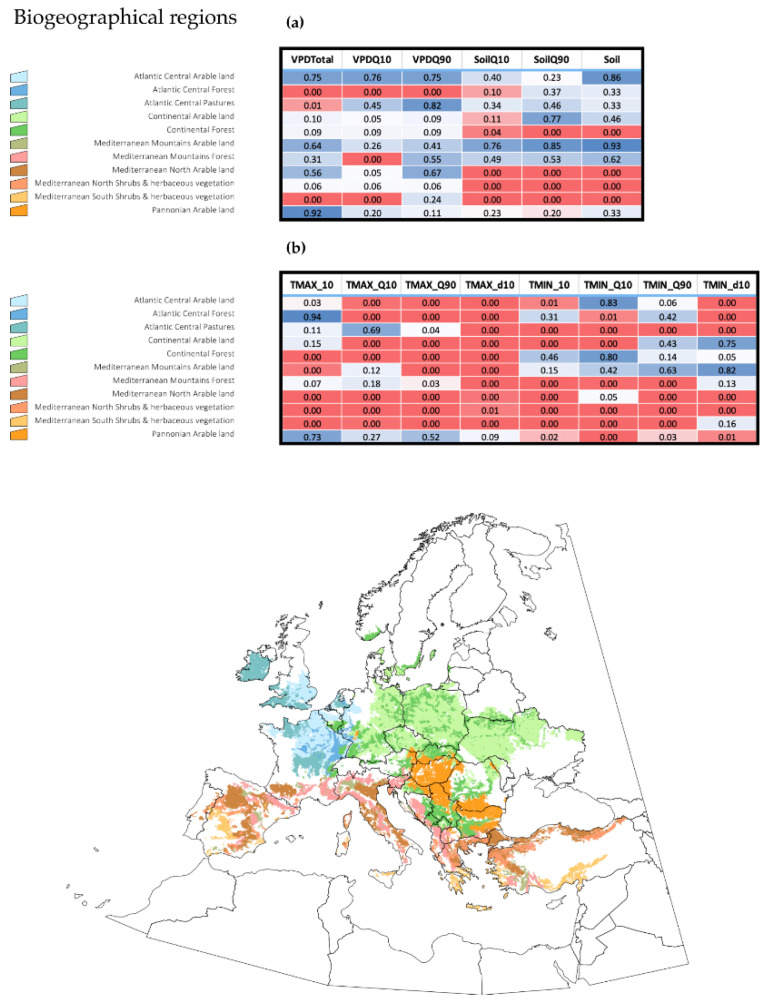
Environmental variables with statistical significance in the changes of environmental suitability for *Hyalomma marginatum* in the period 1970–2018, obtained from a multiple regression among the slope of changes of suitability (dependent variable) and several environmental variables (explanatory variables). The results were calculated separately for each of various selected biogeographic areas, which cover more than 80% of the territory. Two heat maps are associated with the tables in which the “*p*” values for each variable are included: cells in red represent highly significant variables. The abbreviations of the variables are: SoilQ10, SoilQ90, and Soil (percentiles 10 and 90 of the soil moisture and the total annual soil moisture, respectively); VPDTotal (sum of the annual values of daily water vapor deficit), VPDQ10 and VPDQ90 (percentiles 10 and 90 of the of the daily water vapor deficit) in figure (**a**); and TMAX_10 (annual sum of the daily maximum temperature values exceeding of 10 °C), TMAX_Q10 and TMAX_Q90 (percentiles 10 and 90 of the of the daily maximum temperature values), TMAX_d10 (number of days in a year in which maximum temperature exceeded 10 °C); TMIN_10 (annual sum of the daily minimum temperature values exceeding of 10 °C), TMIN_Q10 and TMIN_Q90 (percentiles 10 and 90 of the of the daily minimum temperature values), TMIN_d10 (number of days in a year in which minimum temperature exceeded 10 °C) in figure (**b**).

It is important to derive ecological meaning for the variables driving the changes of ES for *H. marginatum* in Europe. We obtained daily values of several explanatory variables using the complete dataset transformed via harmonic regression (see details in the Methods section) including variables related to maximum and minimum temperature, soil humidity and water vapor deficit. We calculated multiple regressions, e.g., the slopes of ES changes and of the explanatory variables, aiming to determine how both groups of slopes were correlated in the period 1970–2018. The results are displayed in Figure 3, separately for variables related to “water” and those related to “temperature”. Although beyond the scope of the present discussion, it may be noted that quartile 90 temperature and the number of days above a threshold temperature of 10 °C are good indicators of the increase of ES and are the main driving variables in the northern territories of the target region. However, this is not the rule for every territory. Minimum temperature and soil humidity are highly correlated with the slope of ES in some Mediterranean habitats, suggesting that soil humidity has a role in the expansion of ES. It should be noted that minimum temperatures (i.e., quartile 10) are not behind major changes of ES in the northern territories, meaning that warmer minimum temperatures are not correlated with the observed changes in ES for *H. marginatum* at the time scale considered. Instead, the results show that the daily accumulated temperature was the variable which was best able to explain the calculated results. Water vapor deficit, a variable measured well above the ground surface, has little importance in defining the changes in ES for the target species. 

**Figure 4:**
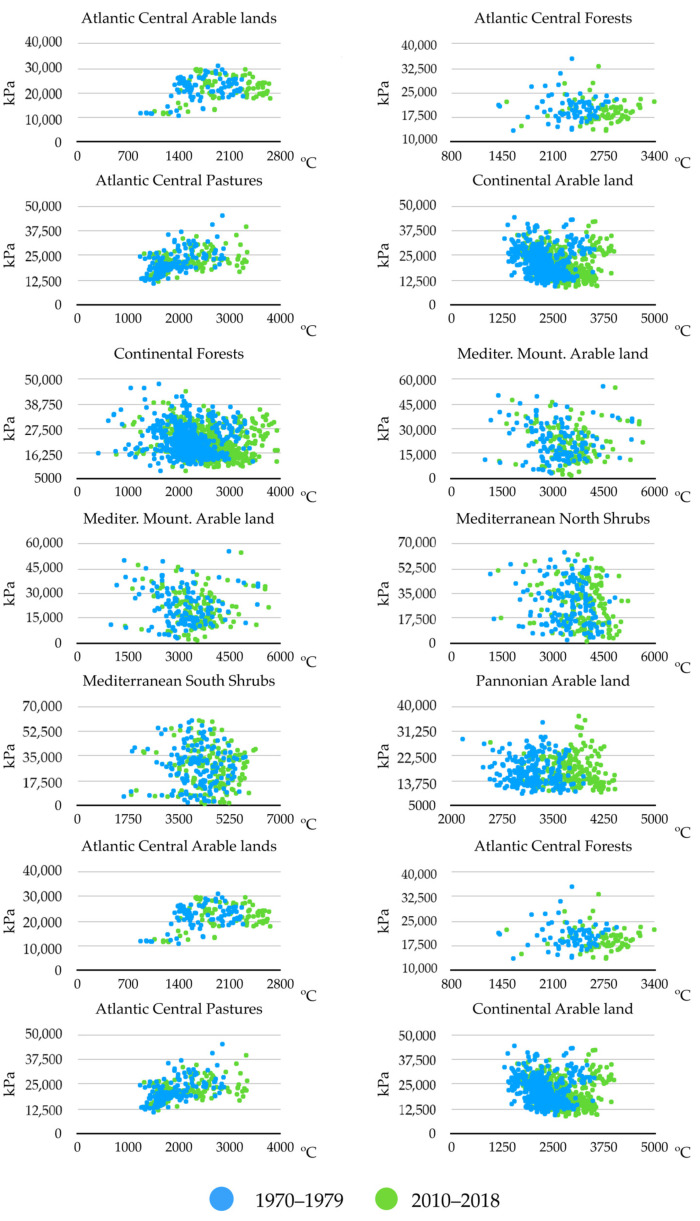
Changes in the sum of daily annual maximum temperature and of solid moisture for selected biogeographical regions. The X axis represents the sum of maximum temperature in degrees Celsius; the Y axis indicates the sum of soil relative humidity in kPa.

Figure 4 displays the changes observed between the years 1970–1979 and 2010–2018, using only two variables, namely, the sum of maximum temperatures and the sum of soil water humidity; these were shown to be significantly involved in multiple regression between the slope of changes of ES and that of trends of the environmental variables. Only these two periods were considered in order to improve the visualization of the charts and provide a summary of the observed changes. The sites included were the same biogeographical categories displayed in Figure 3. Other variables were not included in the charts, and the comments below should be considered a proof-of-concept of: (a) temperature is not the only variable that changes to improve the ES for *H. marginatum*; and (b) each biogeographical region experienced changes of different weather variables, converging into the emergence of suitable niche for the tick. Trends are variable according to the region, but the general view is an increase of the temperature together with a decrease of soil humidity. Changes of soil humidity were negligible in some regions (i.e., Atlantic central regions), in which the most prominent change was the increase of temperature. Continental-type sites mostly observed a decrease of soil humidity. Changes in Mediterranean-type habitats are intermediate, and both variables seemed to act jointly. Pannonian-type habitats observed a clear increase of temperature between the two periods of time.

## 3. Discussion

We sought to evaluate the environmental suitability for *H. marginatum* in the period 1970–2018, its spatial trends and the variables that a have a stronger influence driving the slow but obvious changes observed in the target territory. We used series of climate data and geo-referenced field records of *H. marginatum*. Long series of weather data are difficult to obtain using satellite images, with the most recent beginning only in 2001, i.e., the MODIS series of Earth-orbiting satellites. Satellites have different calibration protocols and a relatively short life; therefore, it is problematic to harmonize data captured by different series of satellites, as this would not result in a long series of comparable environmental data. While we strongly support the use of satellite data for the predictive modelling of spatial distribution of organisms [27], a long series of different weather variables was necessary for this study. For this purpose, we chose the re-analyzed data of the TerraClimate repository, as they provide a long series of data extending back to 1950. As is compulsory for mechanistic modeling, this study is predicated on a set of coordinates recording the known distribution of the tick, together with a set of explanatory variables to generate the models. We previously demonstrated [27] that the use of coefficients resulting from a harmonic regression of the monthly variables produced better predictions than the use of pre-tailored weather variables. The latter provide a good description of the climate across the planet, but they cannot capture the behavior of every organism. Thus, they do not adequately explain the factors regulating the presence of permanent populations of the modeled organism. Since our methods based on harmonic regressions can produce daily values of each trait, this results in a convenient method to derive the necessary variables describing the spatial distribution. In our study, the records of *H. marginatum* were collected approximately in the period 1990–2006; as such, these data were used as the set of training variables averaged to fit the same period.

Traits regulating the colonization of *H. marginatum* have been ascribed to the effect of temperature on development stages [12]. The hypothesis of a critical threshold of accumulated temperature necessary for the spread and colonization of *H. marginatum* [12] was unequivocally confirmed by our results in most of Europe. While the TMax quartile 90 and the number of days with Tmax above a threshold of 10 °C are of importance in almost every ecological region, their impact is spatially different, alone or together with other traits. An unexpected result is the importance of the soil moisture in the driest regions of the continent (i.e., the Mediterranean-type areas). It is interesting to note that this is the area in which *H. marginatum* has been historically recorded. We interpret this result as the joint effect of several variables, with a spatially different relative importance collectively promoting stable niches in the region that is commonly colonized by the tick, resulting in the improvement of its ES.

Another unexpected result is the lack of significant results in the multiple regressions linking the trend of Tmin with ES in many of the coldest parts of the analyzed territory: minimum temperature seems not to be a limiting factor for the suitability of *H. maginatum* in central Europe; this is an area that is commonly considered to be unsuitable for the tick because of the freezing minimum temperatures. Our results show that minimum temperature, as recorded for Europe, should not be considered a factor responsible for high tick mortality, as has been widely suggested [28,29], at least in the time period considered. The trend of ES for *H. marginatum* is driven by changes in variables that have a spatially different relative importance, although the daily sum of Tmax and the number of days above the threshold of 10 °C seem to be the best explanatory variables, at least in the period of time selected in the target territory.

As expected, the water vapor deficit in the air is not a trait impacting long-term changes of the environmental niche of the tick. It is not possible to generalize this finding to other tick species, because of their different ecological requirements. Water vapor deficit, a variable measured well above the ground surface, is of little importance in delineating the changes of ES for *H. marginatum*. This underlines the importance of choosing variables with ecological meaning for every tick species, as opposed to adhering to simplistic, “one size fits all” approaches. However, this trait, together with mean or accumulated rainfall, have been largely used to model the environmental suitability for ticks. We would like to recommend a revision of previously published conclusions regarding the effect of rain or other atmospheric “water” variables on the modeling of the environmental niches of ticks. While NDVI (Normalized Derived Vegetation Index) should be applied when using satellite-derived data, a measure of soil surface moisture seems to be important when using traditional weather traits derived from re-assessments or climate recording stations.

In any case, there is no question that weather trends are giving rise to environmentally suitable conditions for *H. marginatum* in relatively large areas in Europe, a finding that is consistent with numerous reports on areas in the continent in recent years [14,15,16,17,18] and a previous analysis, at a rougher resolution, built on a physiological model [19] exploring developmental and mortality rates. When and where we expect that ES would increase over a critical threshold to allow permanent populations of the tick to become established is difficult to predict, because trends are spatially different. In any case, we hypothesize that environmental changes will not expand northward gradually from the fringes of the currently known tick distribution. We expect that large territories of the target region will simultaneously surpass the critical environmental threshold, and vast domains will become suitable in a few years. This hypothesis is based on the findings reported in this study, pinpointing the combined effects of several restricting variables acting together, an issue which has not been addressed before.

Both critical values of temperature and soil humidity impact the ES for *H. marginatum*, but some variables have a stronger effect according to the biogeographical region, an unexpected result of this study. The joint trend of these traits is altering the ES for the target tick into the optimum at an unprecedented rate in large areas of Europe. Nevertheless, it is necessary to stress that without the adequate density of large vertebrates necessary to feed the adult ticks, permanent populations of *H. marginatum* are highly improbable, even if adequate environmental conditions exist. This is thus a call for active surveillance of the tick, a preliminary step for the adaptation to the impact of this invasive species. 

## 4. Material and methods

### 4.1. Background

We aimed to evaluate the trends of ES of *H. marginatum* in the period 1970–2018 in a territory covering Europe, between 16°W, 28°N and 49°E, 71°N. In this study, we adhered to a strict definition of the environmental niche for *H. marginatum* without considering interactions with hosts. We aimed to evaluate the trend of the climate and the changes of the requisites for tick survival, not to predict the probability of permanent populations of the tick. The environmental niche of an organism is defined as the combination of traits in an area that draw its probability of persistence [27]. To calculate the ES for *H. marginatum*, we used: (i) a set of climate variables between the years 1970–2018; (ii) an evaluation of the ES for the tick using a maximum entropy algorithm, relying on a set of known records of the tick; and (iii) an explicit evaluation of trends in the period and target territory, as well as a solid evaluation of the most important driving variables.

### 4.2. Obtaining Climate Data

Climate data were obtained from the TerraClimate website (http://www.climatologylab.org/terraclimate.html, accessed in January 2020). We chose four re-assessed products, at monthly intervals, namely: (i) maximum temperature (TMax) (ii) minimum temperature (TMin) (iii) water vapor deficit (WVD), and (iv) soil humidity (SH), for the period 1970–2018. These data have a spatial nominal resolution of 4 km. Water vapor deficit is measured at 2 m above the ground. According to previous studies on the ecology of the tick [30], these data should be adequate for defining the environmental niche of *H. marginatum*.

Monthly data were transformed by harmonic regression. This technique was developed by Fourier [31], enabling the decomposition of a time series into a regression defined by sine and cosine, including the first three coefficients of the harmonic regression of each environmental trait are the explanatory variables [27]. This reduces the number of variables which are necessary to describe the niche while retaining complete information about the time series, which is a fundamental in spatial modeling approaches. These coefficients synthetically describe the mean value of each variable for the considered period, the slope in the spring (i.e., how fast or slow is the spring change) and the negative autumn slope (i.e., how fast summer values turn into autumn ones). Once derived to daily values, other environmental variables can be obtained (i.e., the sum of maximum daily temperature). In short, the procedure deconstructs the complete time series into coefficients that describe the original data. It has been demonstrated that this approach produces a better modeling outcome than using interpolated climate data or averaged monthly estimates directly derived from the time series [27]. 

### 4.3. Model Generation 

Several algorithms are available to obtain maps of environmental suitability based on explanatory variables. In this study, we used the algorithm MaxEnt (Maximum Entropy) [32], the efficacy of which has been widely demonstrated [33]. These models need to be “trained” with the known distribution of the species to be modeled, from which the combination of variables that define the probability of a species’ presence is obtained. The records with the coordinates of *H. marginatum* were obtained from a previous compilation [34] and are available at http://dx.doi.org/10.5061/dryad.2h3f2. They are also available as Supplementary Material Figure S1.

To calculate the environmental niche of each species, the “wallace” package [35] for R [36] was used. This package uses the MaxEnt algorithm to obtain the expected ES for the tick and project it onto other time chunks. As explanatory variables, we used the first three coefficients of the Fourier series for TMax, TMin, SH, and WPD. The algorithm was trained with 50% of the records of *H. marginatum*, using the remaining 50% to iteratively check the confidence of the fit. The process was repeated ten times, randomly selecting different training and test sets, obtaining the best possible distribution model. The suitability of each model was verified the use of the area under the curve (AUC) of the test set, because the Akaike information criterion provides unreliable results when applied to a geographical extension [37]. The AUC compares the outcome of each model with the recorded distribution of *H. marginatum*, producing an evaluation of the quality of the model in terms of the similarity of the predicted result with the known distribution of the modeled organism.

Since we wanted to determine the changes of ES for the target tick, we trained the models with environmental data between the years 1990–2006. This is the period for which most of the records were collected, and which should, therefore, best describe the environmental conditions driving its presence. Then, we projected the model into chunks of 10 years to obtain an overview of these changes over the time, covering the periods 1970–1979, 1980–1989, 1990–1999, 2000–2009, and 2010–2018. We also projected the trained model into annual intervals to calculate the trend of ES for *H. marginatum* (see below).

All data resulting from the above calculations were summarized into the biogeographical regions of the target territory. We used the LANMAP2 product [38] that describes the biogeographic characteristics of the European territory, synthesizing the climatic and landscape domains.

### 4.4. Other Calculations

We asked if the change in the ES for *H. marginatum* was correlated with changes of explanatory variables with ecological meaning. It should be noted that the raw coefficients of the harmonic regression are extraordinary descriptors for modeling but lack obvious ecological meaning that can, however, be easily calculated [34]. We aimed to evaluate how the trend of ES for *H. marginatum* correlated with these ecologically meaningful variables. We produced 14 variables from the raw coefficients, namely the average, percentile 10, and percentile 90 of each variable (VPD, SH, TMax, and TMin), as well the daily sum of TMax or TMin above a threshold of 10 °C, and the number of days in a year in which TMax or TMin were above that threshold. These are traits are expected to drive the persistence of *H. marginatum*.

We calculated the slope of a lineal regression for the annual ES of *H. marginatum*, as well as the slope of a lineal regression for the annual values of each variable mentioned in the previous paragraph, done on every single pixel of each raster. We then produced a multiple regression between the ES and the environmental traits. The hypothesis assumes that if changes of slopes of both ES and variables are correlated, *p*-values of variables with statistical significance in the multiple regression will show which variables were changing at the same pace as that of ES in the period 1970–2018. It could thus be assumed that these variables are the main drivers of changes of ES for the target tick.

Multiple regressions were performed separately for each biogeographic region in the target territory, following the denominations of LANMAP2 (see above). The purpose was to demonstrate that different variables have different impacts on the trend of ES of *H. marginatum* according to the landscape features. Since there are literally dozens of biogeographical categories in LANMAP2, we selected the areas of “Atlantic Central Forests”, “Atlantic Central arable land”, “Atlantic Central Pastures”, “Continental arable land”, “Continental Forests”, “Mediterranean mountains arable land”, “Mediterranean mountains Forests”, “Mediterranean northern shrubs”, “Mediterranean southern shrubs”, “Pannonian arable lands” and “Steppic shrubs” as a proof of concept. These categories cover more than 80% of the target territory.

## Data Availability

All the tick distribution data used in this study are available under the Supplementary Materials mentioned above. The complete set of data, from which the distribution of *H. marginatum* was drawn is available at http://dx.doi.org/10.5061/dryad.2h3f2.

## Supplementary Materials

The following are available online at https://www.mdpi.com/2076-0817/10/2/95/s1, Figure S1: The distribution of permanent populations of *Hyalomma marginatum* was obtained from literature data. To note the large numbers of collections in northern Africa, which were included in the models even if not related with the biogeographic regions of Europe.
