# Supplementary figures and images for: Towards New Horizons: Climate Trends in Europe Increase the Environmental Suitability for Permanent Populations of Hyalomma marginatum (Ixodidae)
Source: Pathogens. 2021 Jan 21;10(2):95. doi: 10.3390/pathogens10020095 (PMC7909578; doi:10.3390/pathogens10020095)

## Supplementary material

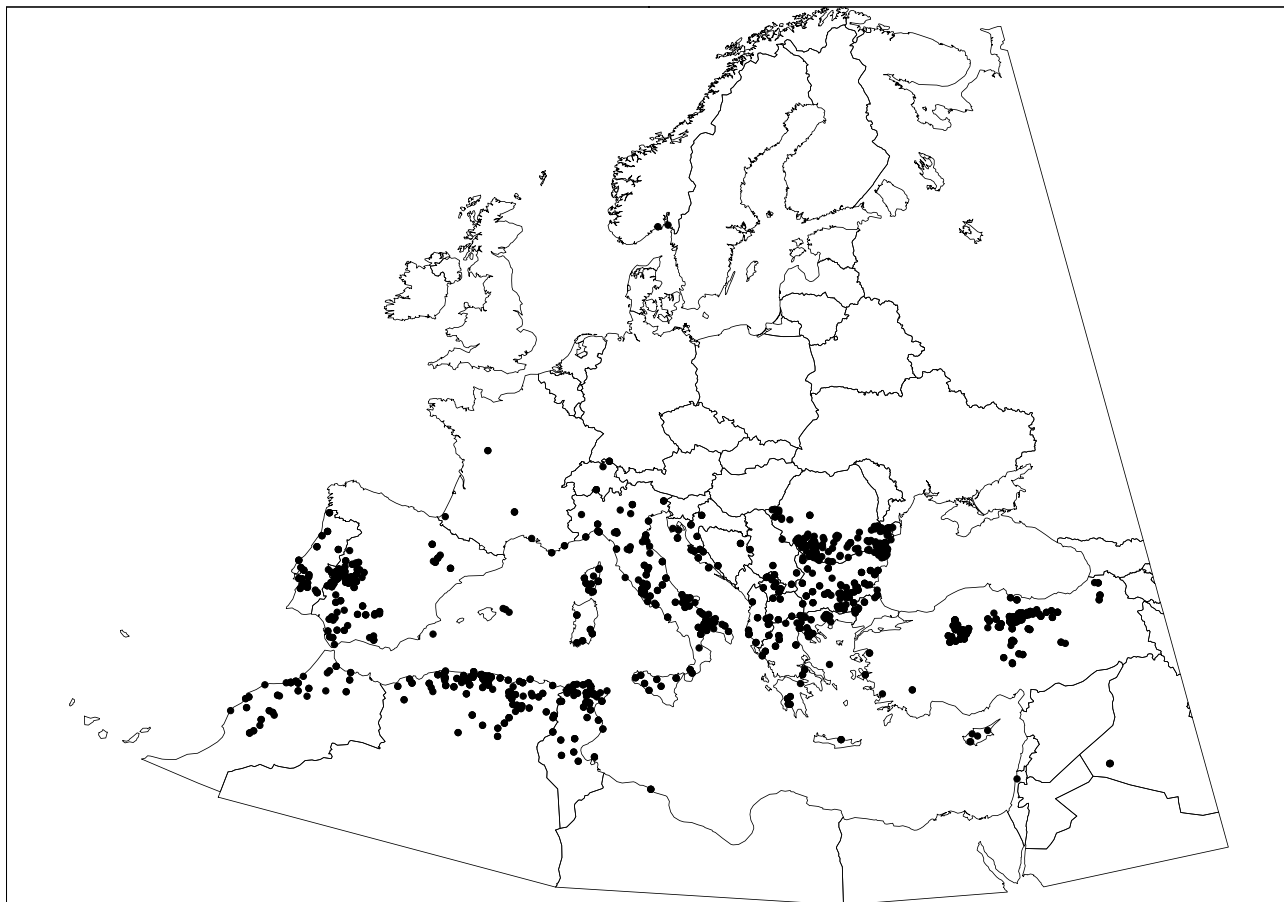

Supplement: Supplementary file 1 [file pathogens-10-00095-s001.pdf]
